# CRISPR-based synthetic biology toolkit development in *Candida viswanthii* and functional analysis of the stress responsive Ena1-like protein

**DOI:** 10.1016/j.synbio.2025.09.021

**Published:** 2025-10-01

**Authors:** Xin-Yue Li, Kai Li, Feng-Li Zhang, Tomohisa Hasunuma, Akihiko Kondo, Lin Zhang, Xin-Qing Zhao, Feng-Wu Bai

**Affiliations:** a Key Laboratory of Microbial Metabolism, School of Life Sciences and Biotechnology, Shanghai Jiao Tong University, Shanghai, 200240, China; b Graduate School of Science, Technology and Innovation, Kobe University, Kobe, Japan; c Engineering Biology Research Center, Kobe University, Kobe, Japan; d SINOPEC Dalian Research Institute of Petroleum and Petrochemicals Co., Ltd., Dalian, 116045, China

**Keywords:** *Candida viswanathii*, CRISPR/Cas9, Iterative editing, Multiplex editing, Multicopy integration, Ena1-like protein g144

## Abstract

Industrial strains from the *Candida* genus have been applied in production of enzymes, biochemicals, and single-cell protein. However, the synthetic biology manipulation tools for *Candida* species remain underdeveloped. In this study, a high-efficiency genome editing strategy for *C. viswanathii* was established by combining the CRISPR/Cas9 and Cre/loxP systems. This approach achieved 100 % editing efficiency and supported rapid iterative editing cycles within 6 days. The system enables iterative genomic modifications and was successfully applied for multiplex editing and multicopy gene integration up to 7 copies. Leveraging this platform, g144, an Ena1-like protein that exhibited differential expression during dodecanedioic acid (DDA) fermentation, was functionally characterized. The results showed that g144 lacks Na^+^ transport activity, but both the disruption and overexpression strains showed increased sensitivity to alkaline pH and Na^+^ stress, as well as a decrease in DDA production. The genome editing toolkit reported here benefits further applications of *Candida* strains for sustainable bioproduction.

## Graphical abstract

**Figure:**
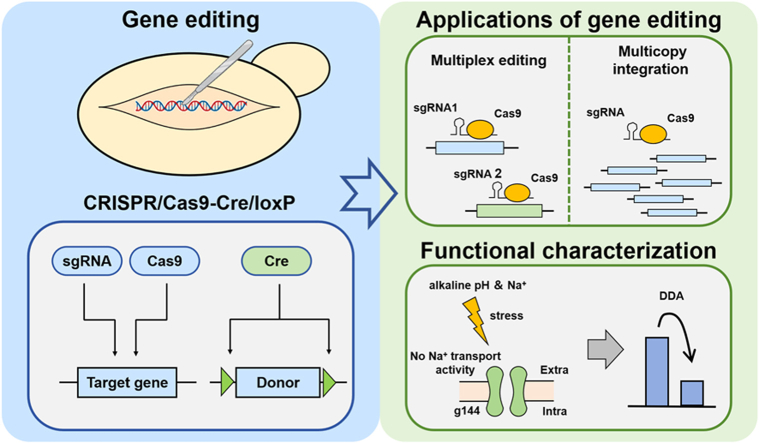

## 1 Introduction

Throughout the history of human civilization, yeast has played a pivotal role in shaping daily life and industrial progress. Approximately a quarter of all yeast species belong to the genus *Candida* [1]. Some *Candida* strains have been used in the bioconversion of agricultural residues (such as lignocellulosic hydrolysates and plant-derived oils), and the sustainable synthesis of enzymes, value-added biochemicals, and single-cell proteins, highlighting their potential in developing resource-efficient bioprocesses [2,3]. Among them, *Candida viswanathii* strains exhibit natural capabilities to utilize both hydrocarbons and fatty acid-based compounds, and have been applied in bioremediation [4] and production of lipase [5] and mid-long chain dicarboxylic acid [6]. Particularly, with the growing demand for high-performance materials such as nylon and resins in recent years, the biotechnological synthesis of DDA, as a more environmentally friendly and sustainable alternative to traditional petrochemical synthesis, has been successfully commercialized by companies such as Cathay Biotech, which utilizes engineered *Candida* strains to produce DDA at an industrial scale [7,8]. In addition, due to the capability of *C. viswanathii* to use xylose as a carbon source, it also has great potential and value for applications in the utilization of renewable lignocellulosic biomass [9]. However, these industrial strains are mainly derived from the screening and long-term domestication of wild-type strains by practitioners, and it is desirable to develop efficient strains for economic production. However, the development of efficient gene editing technology for *C. viswanathii* remains limitedly reported [10].

As a non-conventional yeast, the genus *Candida* presents more difficulties for gene editing than the model yeast *Saccharomyces cerevisiae*. Firstly, some species of the genus *Candida* (such as *C. viswanathii*, *C. albicans*, *C. dubliniensis*, *C. utilis*, *C. tropicalis*, and *C. parapsilosis*) have diploid genomes and do not undergo meiosis. Therefore, each allele must be targeted independently, making the genetic manipulation a difficult process [11]. Secondly, a large number of *Candida* species belong to the CTG clade, in which the CTG codon is reassigned to encode serine instead of leucine [12]. This difference in the codon table leads to the possibility of translation errors or inefficiencies when exogenous genes are expressed. Finally, the predominance of non-homologous end joining (NHEJ) and the weakness of efficient homology-directed repair (HDR) mechanisms in *Candida* strains make precise gene editing difficult [13,14].

All of the above-mentioned factors have meant that gene editing technology for the genus *Candida* is still in its infancy. At this stage, there have been relevant studies of gene editing methods for *C. albicans* [15], *C. parapsilosis* [16], *C. glycerinogenes* [17], and *C. tropicalis* [18,19], using traditional homologous recombination and CRISPR/Cas9 strategies. For *C. viswanathii*, researchers achieved an editing efficiency of 60 % and one-time genome integration of a large DNA fragment (13.6 kb) based on a transient CRISPR-Cas9 system [10]. This is an exciting breakthrough achieved from scratch. However, several technical challenges remain in the genetic manipulation of strains. For instance, large DNA fragments used for transient transformation often exceed 15 kb and are complex to construct. Secondly, despite the urgent demand for various recombinant strains, multiplex editing systems and multi-copy integration in *C. viswanathii* have yet to be reported.

In eukaryotic microorganisms such as yeast, high concentrations of extracellular sodium ions (Na^+^) and alkaline pH can induce stress by disrupting the plasma membrane potential and ionic homeostasis, as well as affecting the function of the proton pump [20,21]. Ena1 (sodium/potassium-exporting P-type ATPase 1), which belongs to the P-type ATPase family, is a key plasma membrane protein that responds to high sodium and alkaline pH stress in *S. cerevisiae* [22,23]. Ena1 expression is affected by a variety of factors, including the environmental conditions (such as Na^+^, K^+^ levels and alkaline pH), signaling pathways (HOG, Ca^2+^-Calcineurin-Crz1, Rim101, and CWI Pathway), and transcription factors [22,24]. This suggests that it is involved not only in ion transport but also in integrating membrane stress signals and maintaining membrane homeostasis [25]. In our previous work, an Ena1-like protein (g144, GenBank accession no. PV865571) was identified in *Candida viswanathii* CICC 33310 (Cv310), with significantly upregulated transcription during DDA synthesis. However, the function of g144 and its links to DDA production remain unexplored.

In this study, a CRISPR/Cas9-Cre/loxP system was developed for efficient gene editing in *C. viswanathii*. Compared to the LINEAR CRISPR-Geminin system, 100 % editing efficiency was achieved with this approach, alongside greater convenience and adaptability for iterative modifications. The editing cycle was reduced to 6 days while maintaining an efficiency of ≥83 %. In addition, multiplex editing was achieved, and genomic integration of 7 copies of *gfp* reporter gene was accomplished. Furthermore, the Ena1-like protein g144 was functionally characterized using this system. The genome editing toolkit reported here benefits further applications of *Candida* strains for sustainable bioproduction.

## 2 Materials and methods

### 2.1 Strains and media

*C*. *viswanathii* CICC 33310 was obtained from the China Center of Industrial Culture Collection (referred to as Cv310 in the following text). The *Escherichia* *coli* DH5α was used as a host for homologous recombination and T4 DNA ligation. All cloning experiments were conducted using *E. coli* DH5α, cultured at 37 °C in Luria-Bertani (LB) medium supplemented with 100 mg/L ampicillin. Yeast-Peptone-Dextrose (YPD) medium was used for the routine cultivation of Cv310. CMf medium (1.18 g/L K_2_HPO_4_, 4.88 g/L KH_2_PO_4_, 3 g/L (NH_4_)_2_SO_4_, 30 g/L glycerol, 3 g/L yeast extract, 6.7 g/L YNB without amino acids, and 0.5 g/L NP-40, pH 5.8) and reaction medium (RM) (0.5 M Tris-HCl buffer, 3 g/L urea, 20 g/L glucose, 6.7 g/L YNB without amino acids, and 0.5 g/L NP-40, pH 8.0) were used for strain culture and DDA production in shake flasks, respectively [10]. The fermentation medium was used for DDA production in shake flasks (30 g/L glucose, 1 g/L yeast extract, 1 g/L NaCl, 5 g/L KH_2_PO_3_, 3 g/L urea, 5 g/L ammonium sulphate, and 5 % (v/v) alkane). The pH of the fermentation medium was maintained at 8.0 with 10 M NaOH. These two media were used to compare the mode of pH regulation and the synthesis of DDA in the presence and absence of Na^+^. The transformants of Cv310 were screened using 100 mg/L Nourseothyrcin (Nrs^R^) or 200 mg/L Hygromycin resistance (Hyg^R^).

### 2.2 Plasmid construction

DNA polymerase and restriction enzymes were purchased from Vazyme and New England BioLabs. Yeast genomic and plasmid DNA were extracted and purified using commercial kits from TIANGEN (Beijing, China). sgRNA sequences were designed using CHOPCHOP (https://chopchop.cbu.uib.no/). Plasmid and primer information are provided in Tables S1 and S2 of the Supporting Information.

The shuttle plasmids pUC57-ARS-Hyg^R^ and pUC57-ARS-Nrs^R^, constructed for gene expression in Cv310, were derived from the universal vector pUC57, incorporating an ARS sequence from *C. albicans* [26]. The codon-optimized Hyg^R^ and Nrs^R^ resistance genes (synthesized by TSINGKE) were driven by the *FBA1* promoter from Cv310 and terminated by the *CYC1* terminator from *S. cerevisiae*. A codon-optimized Cre recombinase cassette, driven by the *ADH2* promoter (Cv310) and terminated by the *CYC1* terminator, was inserted into pUC57-ARS-Nrs^R^ to generate pUC57-ARS-Nrs^R^-Cre, which was used for marker recycling.

To evaluate targeted gene editing efficiency, the plasmid pCashGem-*POX5*-Hyg^R^-LINEAR was constructed to disrupt the *POX5* gene, with the linear backbone and sgRNA amplified from pCashGem-Xyl2-*URA3*-LINEAR (Addgene #174840). The plasmid was then linearized with *Pst*I and *Not*I to yield a single DNA fragment containing the Cas9-hGem/sgRNA cassettes and *POX5* homology arms. To improve homologous recombination efficiency, the *KU70* gene was deleted using pCashGem-*KU70*-Hyg^R^-LINEAR.

The pXY-Cas9 plasmid series was constructed based on the pCT-tRNA system [14] by replacing the *ACT1* promoter of Cas9 with the *TDH1* promoter from Cv310, yielding pXY1-Cas9. The *POX5*-targeting plasmid pXY1-Cas9-POX5 was generated by inserting the sgRNA into *BspQ*I-digested pXY1-Cas9 using T4 ligase (NEB). To optimize sgRNA expression, the *TEF1* promoter was replaced with *PGK1*, *ENO2*, or *TDH3* from Cv310, yielding pXY101-, pXY102-, and pXY103-Cas9-*POX5*, respectively. To evaluate the compatibility of LINEAR platform elements with the classical CRISPR/Cas9 system, Cas9-Geminin and sgRNA cassettes were inserted into the pUC57-ARS-Nrs^R^ backbone to generate pXY2-Cas9 and pXY2-Cas9-*POX5*. Constructs targeting *rDNA*, *POX4*, and *G144* followed the pXY1-Cas9-*POX5* strategy. Donor plasmids pUC57-*POX4*-UP-Hyg^R^-GFP-DW and pUC57-*rDNA*-UP-Hyg^R^-GFP-DW provided repair templates.

To enable multitarget gene editing in *C. viswanathii*, a dual sgRNA cassette was assembled by overlap PCR and inserted into pXY1-Cas9, generating pXY1-Cas9-sgRNA-Plus. The dual-targeting plasmid pXY1-Cas9-sgRNA-*FAT1*-*POX5* was constructed based on pXY1-Cas9-sgRNA-Plus for performance evaluation.

To observe the localization of the g144, pUC57-ARS-HygR-P_*FBA1*_-*G144*-GFP-T_*CYC1*_ was obtained by fusing GFP to g144 through the linker “GDGAGLIN”. To verify whether g144 possesses Na^+^ transport activity, *G144*∗ was codon optimized and chemically synthesized to allow its expression in *S. cerevisiae* AXT3K, and the fragment was subsequently inserted into pYES2 to give pYES2-*G144∗*. To evaluate the effect of g144 on DDA synthesis, the plasmids pUC57-ARS-Hyg^R^-P_*NCP1*_-*G144*-T_*CYC1*_ and pUC57-ARS-Hyg^R^-P_*FBA1*_-*G144*-T_*CYC1*_ were constructed.

### 2.3 Strong promoter screening and multi-copy integration assay

Promoters *TEF2*, *ADH2*, *ENO1*, *ADH1*, *AOX2*, *PGK1*, *TDH1*, and *FBA1* from Cv310 were evaluated for strength using a GFP reporter. Promoter activity and rDNA copy number were assessed by measuring fluorescence intensity normalized to cell density and by quantitative PCR (qPCR), respectively [27,28]. Recombinant strains precultured in YPD were diluted to OD_600_ of 0.2, then washed three times with sterile PBS, and 200 μL aliquots were transferred to 96-well plates. Fluorescence measurements were performed using a microplate reader (Tecan). pXY1-Cas9-*POX4* and pUC57-*POX4*-UP-Hyg^R^-GFP-DW were used for obtaining the control strain Cv310Δ*POX4*-Hyg^R^-GFP.

### 2.4 Genetic transformation of *C. viswanathii*

The transformation protocol of *C. viswanathii* was based on previous work [29] with slight modifications. As for electroconversion of plasmids to *C. viswanathii*, the competent cells (100 μL) were mixed gently with ≈200 ng plasmids (or 2 μg DNA fragments), incubated on ice for 5 min, transferred to a 2 mm cuvette, and electroporated with a pulse of 2.0 kV using MicroPulser Electroporator (Bio-Rad). Subsequently, 1 mL YPD medium was immediately added to the cuvette. The cells were recovered for 2 h, followed by spreading onto selection plates. The plate was incubated at 30 °C for 2–3 days, and colonies were picked for further analysis. The transformation of *S. cerevisiae* was achieved using the LiAc/SS carrier DNA/PEG method [30].

### 2.5 Homologous recombination activity assay in *C. viswanathii*

The homologous recombination activity assay of *C. viswanathii* followed the method of Shao et al. [31]. Three biological replicates were co-transformed with 500 ng each of two PCR products: (i) a dephosphorylated, *Dpn*I-treated pUC57-ARS-Hyg^R^-P_*TDH1*_-GFP-T_*CYC1*_ backbone generated by inverse PCR that disrupts the *gfp* ORF, and (ii) a gfp-restoration fragment bearing 22-bp homology arms. Transformants were plated on YPD agar supplemented with Hyg^R^, incubated for 36 h, and GFP-positive and GFP-negative colonies were counted under a blue-LED transilluminator (US Everbright).

### 2.6 Subcellular localization analysis

To observe the subcellular localization of g144, the yeast cells were cultured in YPD medium containing g144-GFP. After 24 h of growth at 30 °C with shaking at 200 rpm, the cells were washed twice with phosphate-buffered saline (PBS) (pH 7.4). Subsequently, 10 μL of preparations were directly plated on slides and observed at 488 nm with a confocal laser scanning microscope (Nikon Ni-E A1 HD25, Japan).

### 2.7 Na^+^ transport activity assay

For the Na^+^ transport activity assay, functional characterization of proteins in *C. viswanathii* is relatively more challenging due to its diploid nature. In addition, no related transporter detection kit is available in *C. viswanathii*. Therefore, the *G144* sequence was codon-optimized and chemically synthesized before being inserted into the pYES2 to enable expression in the *S. cerevisiae* AXT3K strain (Na^+^ transport activity-deficient strains). The Na^+^ transport activity of the g144 transporter was tested by the commercial Yeast Na^+^ Transport Assay Kit (YH5002-20T, Coolaber Science & Technology, Co., LTD). Cells were cultured to an OD_600_ of 1–2, washed three times with sterile double-distilled water (ddH_2_O), and then diluted to an OD_600_ of 0.2. Spot assays were performed on agar plates supplemented with varying concentrations of sodium ions (0 mM, 50 mM, 100 mM, and 200 mM). The plates were incubated at 30 °C for 2–5 days, and the growth of each strain under different sodium conditions was monitored.

### 2.8 Analytical methods

Cell density was quantified by optical density at 600 nm (OD_600_) using a Multiskan GO microplate reader (Thermo Fisher Scientific, USA). High-performance liquid chromatography (HPLC) was employed to quantify DDA production. The analysis was performed on a Waters e2695 system with a 2489 UV/Vis detector and an Acclaim 120 C18 column (Thermo Scientific). Fermentation supernatants containing DDA were separated and analyzed under the following conditions: the mobile phase consisted of an aqueous solution containing 50 % acetonitrile and 0.2 % phosphoric acid, delivered at a flow rate of 1 mL/min. The column temperature was maintained at 25 °C, and UV–Vis absorption was monitored at a wavelength of 210 nm.

### 2.9 Statistical analysis

Probability analysis to determine statistical significance was performed using the Student's *t*-test with a two-tailed distribution. Compared to the appropriate control strain, P-values less than 0.05 (P < 0.05) were considered significant in this study.

## 3 Results

### 3.1 Identification of strong promoters in Cv310

**Fig. 1:**
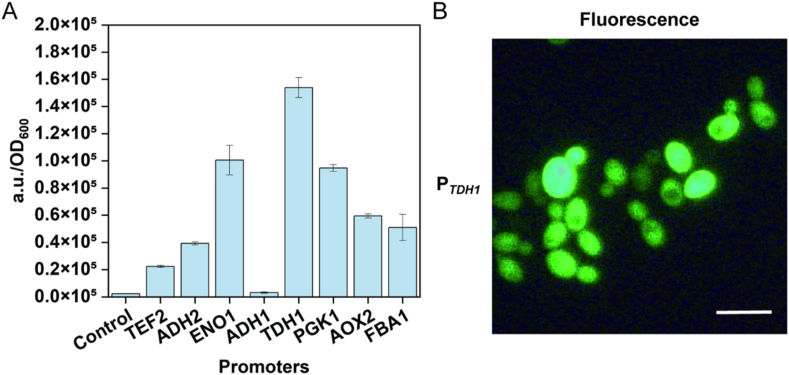
Screening for endogenous strong promoters in Cv310. (A) Characterization of the promoter strength of Cv310 by the fluorescence intensity of GFP. (B) Fluorescence observation of Cv310-pUC57-ARS-Hyg^R^-P_*TDH1*_-GFP-T_*CYC1*_ under a fluorescence microscope. Images were processed using ImageJ.

Endogenous promoter elements play an important role in gene editing and molecular manipulation and help to solve the problems of poor exogenous promoter compatibility and unstable expression [32,33]. Most promoter studies in the genus *Candida* have focused on *C. albicans* [34,35], and there is still little reported about the promoters of *C. viswanathii*. Eight promoters commonly used in yeast (P_*TEF2*_, P_*ADH1*_, P_*ADH2*_, P_*AOX2*_, P_*ENO1*_, P_*FBA1*_, P_*PGK1*_, P_*TDH1*_) were selected from the Cv310 genome, and *gfp* was used as a reporter gene to characterize the strength of these promoters (Fig. 1A). The strongest fluorescence levels were obtained when the *TDH1* promoter was used (Fig. 1B). In addition, P_*PGK1*_ and P_*ENO1*_ also had high expression intensities. This will provide a suitable set of components for the construction of gene editing systems.

### 3.2 Implementation of the LINEAR CRISPR-Geminin platform in *C. viswanathii*

**Fig. 2:**
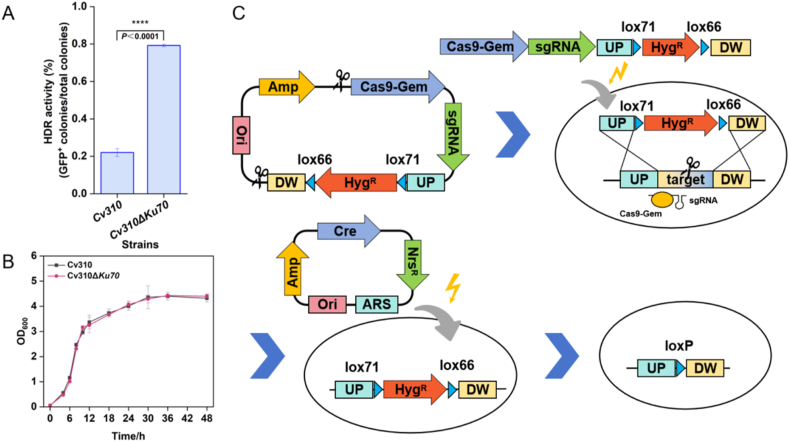
Enhancement of the homologous recombination capability of Cv310 and construction of a gene editing system based on the LINEAR CRISPR-GEMININ platform. (A) Homologous recombination capacity test of Cv310 and Cv310Δ*KU70*. (B) Growth curve of Cv310 and Cv310Δ*KU70*. (C) Scheme of gene editing process using the LINEAR CRISPR-GEMININ platform.

Based on homologous recombination assays, Cv310, a nonconventional yeast, exhibits an NHEJ-proficient background with a homologous recombination efficiency of only ∼22 % (Fig. 2A), which is significantly lower than that of *S. cerevisiae* [31]. Therefore, it is essential to develop an efficient and precise gene editing method suitable for NHEJ-proficient backgrounds. The LINEAR CRISPR-Geminin platform (pCashGem-Xyl2-*URA3*-LINEAR, available from Addgene, 174840) developed by Shao et al. [31] was used. Based on this plasmid, the knockout plasmid pCashGem-*POX5*-Hyg^R^-LINEAR was constructed and achieved 100 % editing efficiency for *POX5* (Table 1). This represents the first successful application of the LINEAR CRISPR-Geminin platform in *C. viswanathii*. Its successful implementation also complements the existing genetic toolbox for *Candida* species. For iterative editing, lox 66 and lox 71 sites were designed to flank the Hyg^R^ gene, and the Cre/loxP system was employed to recycle the selection marker (Fig. 2C).

**Table 1:** Gene editing efficiency of different systems.

Strains	Exogenous DNA constructs		Clones	Efficiency %
	Plasmids	Donor		
Cv310	–	Cas9-Gem-*POX5*-sgRNA-UP-Hyg^R^-DW	10^1^–10^2^	100
	pCT-tRNA-*POX5*	Donor-*POX5*-UP-DW	10^1^–10^2^	0
	pXY1-Cas9-*POX5*	Donor-*POX5*-UP-DW	10^1^–10^2^	10
	pXY101-Cas9-*POX5*	Donor-*POX5*-UP-DW	10^1^–10^2^	0
	pXY102-Cas9-*POX5*	Donor-*POX5*-UP-DW	10^1^–10^2^	0
	pXY103-Cas9-*POX5*	Donor-*POX5*-UP-DW	10^1^–10^2^	0
	pXY2-Cas9-*POX5*	Donor-*POX5*-UP-DW	10^1^–10^2^	0
	–	Donor-*POX5*-UP-Hyg^R^-DW	0 ∼10^1^	33
	pXY1-Cas9-*POX5*	–	10^1^–10^2^	0
	pXY1-Cas9-*POX5*	Donor-*POX5*-UP-Hyg^R^-DW	10^1^–10^2^	67
Cv310Δ*KU70*	–	Donor-*POX5*-UP-Hyg^R^-DW	0 ∼10^1^	83∼100
	pXY1-Cas9-*POX5*	–	0	n.a.
	pCT-tRNA-*POX5*	Donor-*POX5*-UP-Hyg^R^-DW	10^1^–10^2^	0
	pXY1-Cas9-*POX5*	Donor-*POX5*-UP-Hyg^R^-DW	10^2^–10^3^	100
	pXY101-Cas9-*POX5*	Donor-*POX5*-UP-Hyg^R^-DW	10^2^–10^3^	91∼100
	pXY102-Cas9-*POX5*	Donor-*POX5*-UP-Hyg^R^-DW	10^2^–10^3^	66∼100
	pXY103-Cas9-*POX5*	Donor-*POX5*-UP-Hyg^R^-DW	10^2^–10^3^	72∼100
	pXY1-Cas9-sgRNA-*FAT1*-*POX5*	Donor-*POX5*-UP-Hyg^R^-DW + Donor-UP-Hyg^R^-DW	10^1^–10^2^	10∼25

### 3.3 Establishment of a CRISPR/Cas9-Cre/loxP genome editing system in *C. viswanathii*

Although the LINEAR CRISPR-Geminin platform achieved high editing efficiency, its low plasmid construction efficiency became a limiting factor, driving us to explore alternative methods. Phylogenetic studies have shown that *C. viswanathii* and *C. tropicalis* are closely related [36]. Therefore, the *C. tropicalis*-based pCT-tRNA system was tested for application in Cv310. However, the results of co-transformation of Cv310 by pCT-tRNA-*POX5* and Donor-*POX5*-UP-DW showed that it was difficult to obtain positive transformants (Table 1).

**Fig. 3:**
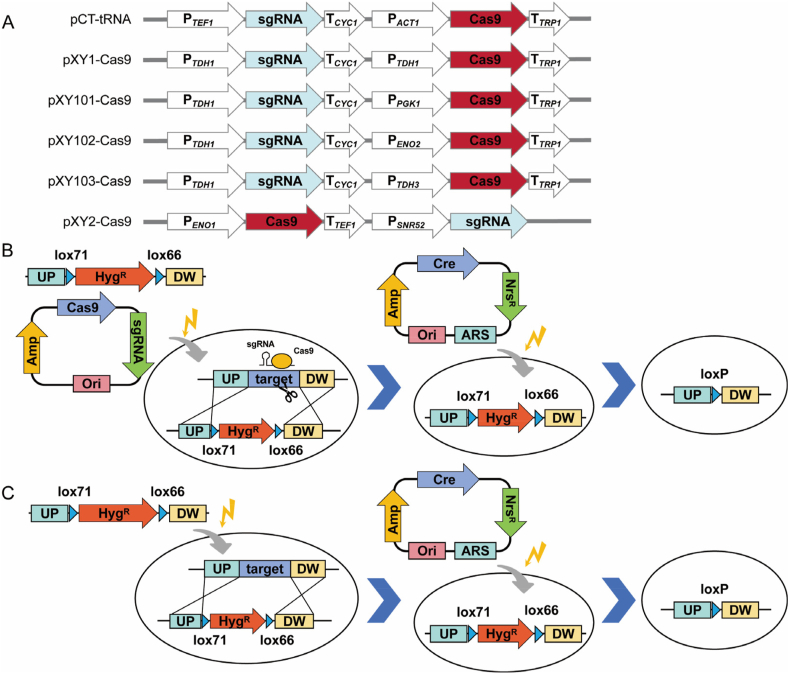
Gene editing based on the CRISPR-Cas9-Cre-loxP and HDR-Cre-loxP systems. (A) Plasmids of the pXY-Cas9 series; (B) The CRISPR-Cas9-Cre-loxP system was implemented through co-transformation of the knockout plasmid and the donor DNA fragments; (C) The HDR-Cre-loxP system utilized transformation of donor DNA fragments alone to accelerate the iteration cycle.

The promoters of the Cas9 and sgRNA cassettes in the pCT-tRNA system were replaced with strong promoters derived from Cv310, resulting in a series of plasmids including pXY1-Cas9, pXY101-Cas9, pXY102-Cas9, and pXY103-Cas9 (Fig. 3A). In addition, the CRISPR/Cas9-hGem cassette and sgRNA cassette derived pCashGem-Xyl2-*URA3*-LINEAR were inserted into pUC57-ARS-Nrs^R^, obtained the pXY2-Cas9. Unfortunately, the transformants were mostly false positives (Table 1), making it difficult to obtain correct transformants that were precisely edited. This may result from the strong NHEJ repair capacity of Cv310, where CRISPR/Cas9-induced double-strand breaks (DSBs) are preferentially repaired through NHEJ rather than homologous recombination. Therefore, the *KU70* of Cv310 was knocked out using the LINEAR CRISPR platform. The result shows that the HDR activity of Cv310Δ*KU70* was improved to 79 % (Fig. 2A). The deletion of *KU70* did not significantly affect the growth of the strain in YPD medium (Fig. 2B), underlining that the suppression of NHEJ-related proteins is an effective strategy to increase HDR rates.

To determine whether inefficient Cas9 activity contributed to false-positive transformants, pXY1-Cas9-POX5 was introduced into both Cv310 and Cv310Δ*KU70*. The results showed that while some clones appeared on the Cv310 transformation plate, no colonies grew on the Cv310Δ*KU70* plate (Fig. S1). This suggests that in strains with active NHEJ, DSBs induced by Cas9 can be repaired through NHEJ, allowing cells to survive. Conversely, it is difficult to obtain transformants. These results indicate that Cas9 is sufficiently effective in inducing lethal DSBs and that the presence of false-positive clones is likely due to the high NHEJ activity in Cv310.

**Fig. 4:**
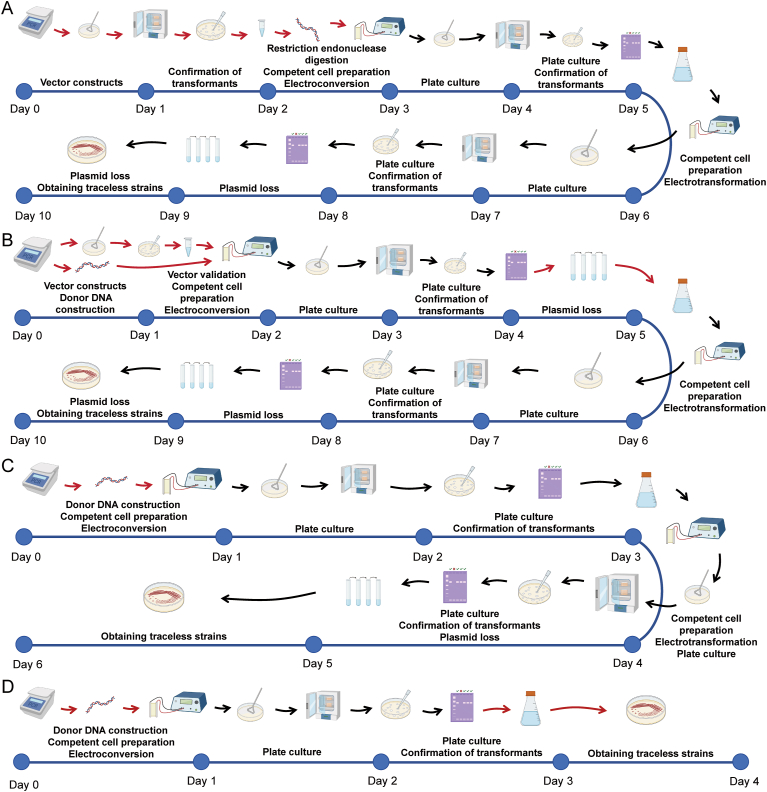
The workflow and iterative editing cycles for the different gene editing strategies. Gene editing cycle and workflow based on (A) the LINEAR CRISPR-Geminin platform, (B) the CRISPR/Cas9-Cre/loxP system, and (C) the HDR-Cre/loxP system. The red arrows indicate the differences between the different strategies.

In order to improve the screening efficiency, the Hyg^R^ screening marker in the Donor fragment was placed. The results showed that the gene editing efficiency of the pXY series of plasmids was significantly improved. Among them, 100 % gene editing efficiency could be achieved when using pXY1-Cas9 (Table 1). It shows that the CRISPR/Cas9 system-based classical strategy was effective and valuable for industrial applications (Fig. 3B). Notably, our strategy reliably achieves a 10-day iterative editing cycle under standard experimental conditions, matching the cycle length reported for the LINEAR CRISPR-Geminin platform under optimal conditions (Fig. 4A and B).

Due to the significant enhancement of homologous recombination (HDR) efficiency in the strain, gene editing performance was evaluated without employing a knockout plasmid, using only the donor DNA fragment for transformation. Remarkably, gene editing efficiencies of 83–100 % were achieved under these conditions (Table 1). This is highly encouraging, as it significantly reduces the reliance on knockout plasmid construction (Fig. 3C), greatly simplifies the workflow, and shortens single-gene editing cycles from 4–5 d to 3 d, and iterative editing cycles from 10 d to 6 d, respectively (Fig. 4C).

### 3.4 CRISPR/Cas9-Cre/loxP-mediated multi-gene editing system in *C. viswanathii*

**Fig. 5:**
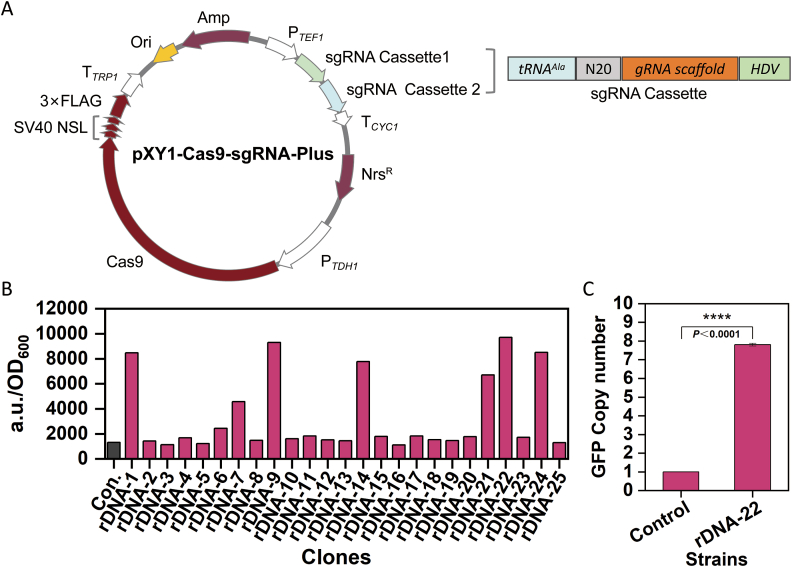
Multiplex editing and multi-copy integration based on *C. viswanathii*. (A) The plasmid profile of pXY1-Cas9-sgRNA-Plus. The plasmid shares the main features of the pCT-tRNA system. In particular, the Cas9 promoter was replaced by the Cv310 *TDH1* promoter, and two sgRNA cassettes were driven by the *TEF1* promoter. (B) Copy number assay for multi-copy integration. The control strain was Cv310Δ*KU70*Δ*POX4*-Hyg^R^-GFP. (C) Relative quantification of *gfp* gene copy number by qPCR. qPCR was performed to estimate the relative *gfp* gene copy number in the engineered strain compared to the control strain (Cv310Δ*KU70*Δ*POX4*-Hyg^R^-GFP) containing a single copy of *gfp* gene integration. The ΔΔCt method was used for analysis, with *ACT1* as the internal reference gene.

To further accelerate the construction of *C. viswanathii* cell factories, the multiplex editing manipulation in Cv310 was explored. The plasmid pXY1-Cas9-sgRNA-Plus, containing two sgRNA expression cassettes, was constructed (Fig. 5A) and used to simultaneously target the very-long-chain fatty acid transport protein gene *FAT1* and *POX5*, achieving up to 25 % dual-target editing efficiency (Table 1). The remaining transformants exhibited recombination events at only one of the two targets. This is the first successful application of multiplex genome editing in *C. viswanathii*, indicating the feasibility of simultaneous multi-gene targeting and providing a strong foundation for the continued optimization of genome engineering strategies.

### 3.5 Feasibility assessment of multicopy integration at the rDNA locus in *C. viswanathii*

To evaluate the feasibility of multicopy genomic integration at the 18S rDNA locus in *C. viswanathii*, the pXY1-Cas9-rDNA and the donor rDNA-UP-Hyg^R^-GFP-DW were co-transformed into the Cv310*ΔKU70*. Cv310*ΔKU70*Δ*POX4*-Hyg^R^-GFP was used as a control strain, and 25 transformants were randomly selected for fluorescence detection. The results showed that approximately 70 % of the transformants exhibited GFP fluorescence levels comparable to the control strain, indicating that only a single copy of *gfp* was integrated. However, some transformants (such as rDNA-9) displayed fluorescence intensities up to seven times higher than the control, suggesting that up to seven copies of *gfp* may have been integrated into their rDNA locus [37] (Fig. 5B). To further determine the copy number of *gfp*, the copy number of the rDNA-22 transformant was examined using quantitative PCR (qPCR). The results showed that 7–8 copies of GFP had been successfully integrated (Fig. 5C). This work presents the first application of rDNA multicopy integration in *C. viswanathii*, demonstrating its feasibility and providing a basis for future metabolic engineering efforts.

### 3.6 Functional characterization of Ena1-like protein g144 using CRISPR/Cas9-Cre/loxP genome editing in *C. viswanathii*

In previous work, a gene (*G144*) in Cv310 was identified that shows significant transcript upregulation during DDA synthesis (data not shown). To analyse the relationship between g144 and DDA production, the function of g144 was characterised.

**Fig. 6:**
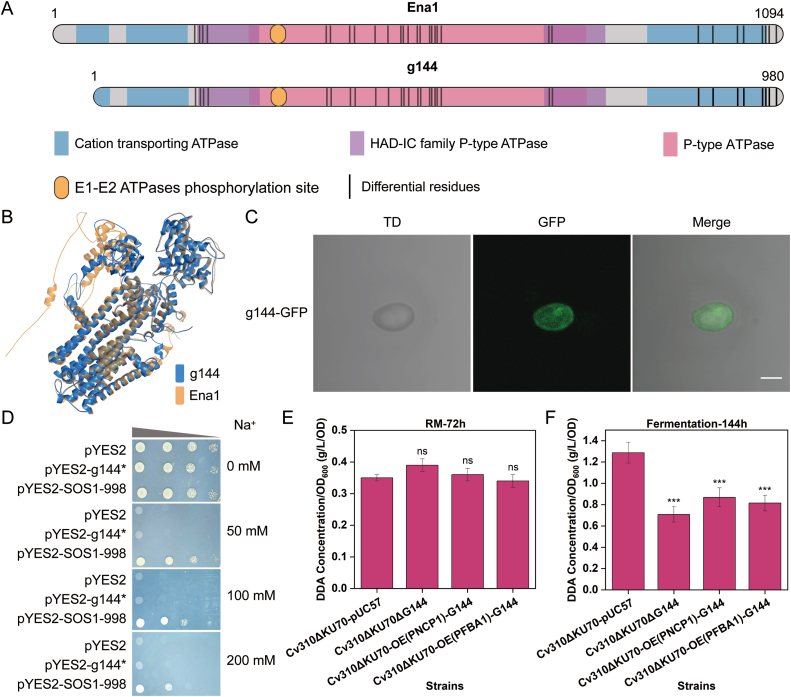
Functional characterization of g144 in *C*. *viswanathii*. (A) Amino acid sequence and structural analysis of Ena1 and g144 (Interpro, https://www.ebi.ac.uk/interpro/). (B) Comparison of the structures of Ena1 and g144. The structure of Ena1 was obtained from Uniprot (https://www.uniprot.org/), and the structure of g144 was obtained using Alphafold3 (https://studio.hpc.sjtu.edu.cn/). The yellow and blue colors represent the structures of Ena1 and g144, respectively. Structural superimposition and RMSD calculation of g144 and Ena1 were conducted in PyMOL using the align command, which performs sequence-based alignment and calculates RMSD over matched residues. (C) Subcellular location of g144. (D) Na^+^ transport activity assay of g144. Strains carrying plasmids pYES2 and pYES2-SOS1-998 were used as negative and positive controls, respectively. Salt-overly-sensitive 1 (SOS1) is a unique electroneutral Na^+^/H^+^ antiporter at the plasma membrane of higher plants (Ena1, UniProt ID: A0A367XZ75, has not been functionally studied.). (E–F) DDA production assay of g144 recombinant strains per unit biomass. Fermentation pH was maintained in the RM system by Tris-HCl buffer and in the fermentation medium system by timed replenishment of NaOH (12 h intervals). The *NCP1* and *FBA1* promoters were used to evaluate the effect of g144 on DDA synthesis under low and high levels of transcription intensity.

Initially, phylogenetic analysis based on the g144 amino acid sequence revealed its highest similarity (97 %) to the P-type Na^+^ transporter Ena1 (Uniprot ID: A0A367XZ75) from *C. viswanathii* ATCC 20962 (Figs. S2–S3) [38,39]. Ena1 is known to play a crucial role in sodium detoxification mechanisms [40]. Despite the high similarity between these two proteins, g144 was found to contain only 9 transmembrane helices (TMHs), compared to the 10 TMHs in Ena1 (Fig. S4), and the deletion of this TMH (114aa) disrupts the cation-transporting ATPase domain at the N-terminal end (Fig. 6A and B). In addition, there are 30 different amino acids between g144 and Ena1 in the cation-transporting ATPase, HAD-IC family P-type ATPase, and p-type ATPase domains. Structural analyses of g144 and Ena1 showed that the root mean square deviation (RMSD) was only 0.689 Å (measured using PyMOL). These results suggest that g144 is structurally very similar to Ena1, demonstrating a high degree of structural conservation.

Ena1 has been reported to localize to the plasma membrane [24]. However, the subcellular localization of g144 requires further investigation due to a partial deletion in its N-terminal domain. First, the g144 protein sequence was analyzed. Protter (https://wlab.ethz.ch/protter/) [41] predicted a 14 aa signal peptide at the N-terminus of g144 (Fig. S4), strongly suggesting its potential localization to the plasma membrane. Second, the g144-GFP fusion protein was expressed in Cv310 and analyzed in the recombinant strain. The results revealed ring-shaped GFP fluorescence along the cell periphery, with weak and non-diffuse cytoplasmic fluorescence [42], indicating that g144 is also localized to the plasma membrane (Fig. 6C).

To investigate whether g144 still possessed Na^+^ transport activity, the g144∗ (by chemically synthesising codon-optimized g144 to enable its expression in *S. cerevisiae*) was expressed in the Na^+^ transporter-deficient strain *S. cerevisiae* AXT3K. The results showed that the g144∗ recombinant strain did not grow on plates containing various concentrations of Na^+^ (Fig. 6D). This indicates that g144 lacks Na^+^ transport activity, which may be due to differences with Ena1 in its cation-transporting ATPase domain and specific amino acid residues. Furthermore, this suggests that, despite its high similarity to Ena1, g144 does not function as a conventional Na^+^ transporter and may possess other physiological roles.

The synthesis of DDA in yeast is pH-dependent [43,44]. Consequently, during industrial DDA production, large amounts of NaOH are usually added to maintain an alkaline pH [45]. Therefore, the effect of *G144* knockout and overexpression on DDA synthesis was evaluated in two culture systems (RM and fermentation medium). The results showed that in RM (pH maintained by Tris-HCl, pH 8.0, Na^+^ free), there was no significant difference in DDA production per unit biomass between both *G144* knockout and overexpression and control strains (Fig. 6E). In contrast, in fermentation medium conditions (pH 8.0 maintained by 10 M NaOH), there is a significant decrease in DDA production per unit biomass was observed for both *G144* knockout and overexpression strains (Fig. 6F). One report showed that the expression of Ena1 was strongly increased by exposure to high Na^+^ concentrations or alkaline pH [46]. Disruption of Ena1 results in impaired Na^+^ efflux capacity and is highly sensitive to Na^+^ and alkaline pH stress conditions [47]. Therefore, it was expected that knockout of g144 would result in a strain sensitive to alkaline pH and Na^+^. However, overexpression of g144 driven by different promoters also showed a significant decrease in DDA production with no significant change in biomass (Fig. S5), suggesting that the decrease in DDA production is not due to toxicity of g144, but may be due to a stress response involving g144 under conditions of elevated Na^+^ concentration and alkaline pH.

## 4 Discussion

*C. viswanathii* has been widely utilized in chemical synthesis applications. However, genetic manipulation of this yeast remains challenging due to its CTG clade identity and diploid nature, both of which hinder metabolic engineering and cell factory development. Previous efforts by Hu et al. [10] and Shao et al. [31]. Established transient transformation-based CRISPR/Cas9 systems for this organism. Notably, the LINEAR CRISPR-Geminin platform of Shao et al. [31] incorporated Geminin to improve HDR efficiency in NHEJ-proficient strains [48,49]. Theoretically, restricting genome editing to the S/G2 phases should increase HDR rates [50]. Geminin prevents DNA rereplication by binding to the licensing factor Cdt 1, and is ubiquitinated and degraded by the APC/Cdh1 complex during G1 phase. In the Cas9-Gem fusion, the ubiquitination core sequence (amino acids 1–110) of Geminin was used to induce G1 phase degradation of Cas9, resulting in improved HDR efficiency [51,52]. Based on this strategy, the LINEAR CRISPR-Geminin platform achieved 100 % editing efficiency in *Scheffersomyces stipitis*, a related CTG-clade species.

The platform was subsequently applied to *C. viswanathii*, where similarly complete editing efficiency was obtained in Cv310. However, low ligation efficiency, poor large plasmid propagation, and PCR-induced mutations limited the platform's reliability and hindered its use in rapid or high-throughput genome editing.

To develop a more rapid and efficient genome editing system, we adopted a classical co-transforming strategy involving knockout plasmids and Donor DNA fragments. Building upon the pCT-tRNA system of *C. tropicalis* [14], we engineered a CRISPR/Cas9-Cre/loxP system through promoter optimization and enhanced selection pressure, achieving 100 % gene editing efficiency in *C. viswanathii*. This optimized platform enables iterative genome modifications with remarkable speed-6 days per editing cycle, and as little as 3 days for single-gene edits-without compromising efficiency or reliability. A recent report based on a transient transformation strategy successfully achieved multiplex editing in *C. tropicalis*, with a dual-target editing efficiency of 32 % [19]. In this study, we assessed the multiplex editing efficiency of the CRISPR/Cas9-Cre/loxP system in *C. viswanathii*, achieving dual-target editing efficiencies of up to 25 %. While this result is encouraging, it also indicates potential for further optimization. We hypothesize that co-transforming the knockout plasmid and the two separate donor fragments into the same cell is challenging, thereby reducing gene editing efficiency (as transient transformation strategies typically introduce only a single DNA fragment). To address this, we propose that linking the two donor DNA fragments could improve multiplex editing efficiency by enabling co-transformation of the extended donor fragment together with the knockout plasmid.

In eukaryotic organisms, rDNA loci serve as prime targets for multicopy gene integration due to their high genomic copy number [[53], [54], [55]]. Previous studies have demonstrated the integration of exogenous xylanase into the *C. utilis* genome using an 18S rDNA shuttle plasmid, achieving an enzyme activity of up to 60 IU/mL [56]. Similarly, a heterologous lactate dehydrogenase was expressed in *C. amazonensis*, and 44 g/L of lactate was obtained with a yield of 0.85 g lactate/g xylose [57]. Building on these works, the potential of the CRISPR/Cas9-Cre/loxP system for multicopy integration was evaluated. Seven *gfp* copies were successfully integrated into the 18S rDNA locus, which confirms the feasibility of rDNA-targeted multicopy integration in *C. viswanathii* and establishes a robust platform for high-performance cell factory construction. For future optimization, the homologous recombination capacity of the strain could be enhanced by overexpressing genes such as *RAD52* [58], thereby improving the efficiency of multi-copy integration. Furthermore, the promoter of Hyg^R^ in the linear donor fragment could be replaced with a weaker promoter to indirectly increase copy numbers through selection pressure, which has shown to be successful in the previous studies in *Yarrowia lipolytica* [59].

To further validate the effectiveness of the CRISPR/Cas9-Cre/loxP system in *C. viswanathii*, we characterised the function of g144, an Ena1-like protein identified in the Cv310. Despite its overall structural similarity to Ena1 and its localization to the plasma membrane, structural analysis revealed that g144 lacks part of the conserved domain of typical cation-transporting ATPases and exhibits a few amino acid differences. Functional assays for Na^+^ transport activity demonstrated that expression of g144 in a Na^+^ transporter-deficient background failed to restore function, suggesting that g144 does not possess active sodium transport capability.

In order to assess its impact on DDA fermentation, we constructed both *G144* knockout and overexpression strains and performed DDA fermentation under two alkaline cultivation conditions. Notably, no significant difference in DDA production was observed among the strains under the Tris-HCl condition (Na^+^ free) (Fig. S5A–B). However, under NaOH-based pH control, both the deletion and overexpression of g144 led to a significant reduction in DDA titer (Fig. S5C–D). While deletion of g144 was expected to cause strain stress in response to Na^+^ and alkaline pH [47], the overexpression strain's phenotypic sensitivity was unexpected. It suggests that g144 may be dosage-sensitive and subject to fine regulatory control.

It has been shown that Ena1 is regulated by multiple stress signals and functions alongside membrane lipid remodeling [60], pH-sensing pathways (e.g., Rim101) [61], and transmembrane stress sensors in the yeast response to alkaline stress [23]. Therefore, we propose a hypothesis that g144 functions as a non-canonical, membrane-associated, dosage-sensitive factor rather than as an ion pump. Deletion of g144 compromises the ability to adapt to Na^+^/alkaline stress [47], whereas the overexpression may lead to sensitivity due to the toxicity to the cell membrane and cellular metabolism [62]. Consequently, both g144 knockout and overexpression converge on reduced DDA productivity per biomass under NaOH (high-Na^+^/alkaline) conditions, whereas in Tris-HCl (Na^+^ free) conditions, upstream signaling may be only weakly induced and differences between strains are minimal. This model explains the distinct DDA yields observed across different media.

## 5 Conclusion

This study establishes an efficient genome editing toolkit that significantly advances synthetic biology and cell factory development in *C. viswanathii*, which can also be adopted for engineering other *Candida* species. Furthermore, the functional characterization of the Ena1-like protein g144 offers new insights into its potential role in regulating alkaline stress for future functional genomic studies of *Candida* species.

## CRediT authorship contribution statement

**Xin-Yue Li:** Writing – original draft, Visualization, Data curation. **Kai Li:** Writing – review & editing. **Feng-Li Zhang:** Writing – review & editing. **Tomohisa Hasunuma:** Writing – review & editing. **Akihiko Kondo:** Writing – review & editing. **Lin Zhang:** Writing – review & editing. **Xin-Qing Zhao:** Writing – review & editing, Funding acquisition. **Feng-Wu Bai:** Writing – review & editing.

## Funding

This work was financially supported by the National Key Research and Development Program (No. 2022YFE0108500).

## Declaration of competing interest

The authors declare the following financial interests/personal relationships which may be considered as potential competing interests: Lin Zhang is currently employed by SINOPEC Dalian Research Institute of Petroleum and Petrochemicals Co., Ltd.

## Abbreviations

Cv310: *Candida viswanathii* CICC 33310
LB: Luria-Bertani
YPD: Yeast-Peptone-Dextrose
NHEJ: non-homologous end joining
HDR: homology-directed repair
DDA: Dodecanedioic acid
Nrs^R^: Nourseothyrcin
Hyg^R^: Hygromycin resistance
RM: reaction medium
PBS: phosphate-buffered saline
Cre: Cyclization Recombination Enzyme
Pox5: Acyl-Coenzyme A oxidase 5
Pox4: Acyl-Coenzyme A oxidase 4
Fat1: very long-chain fatty acid transporter
qPCR: quantitative PCR
Ena1: sodium/potassium-exporting P-type ATPase 1
RMSD: root mean square deviation
rDNA: ribosomal DNA
TMHS: transmembrane helices
HPLC: High-performance liquid chromatography
TD: Transmitted Detector
