# Supplementary material for: CRISPR-based synthetic biology toolkit development in Candida viswanthii and functional analysis of the stress responsive Ena1-like protein
Source: Synth Syst Biotechnol. 2025 Oct 1;11:298–308. doi: 10.1016/j.synbio.2025.09.021 (PMC12590291; doi:10.1016/j.synbio.2025.09.021)
Supplement: Multimedia component 1 [file mmc1.docx]

## CRISPR-based synthetic biology toolkit development in *Candida viswanthii* and functional analysis of the stress responsive Ena1-like protein

Xin-Yue Li^a^, Kai Li^a^, Feng-Li Zhang^a^, Tomohisa Hasunuma^b^, Akihiko Kondo^b^, Lin Zhang^c^, Xin-Qing Zhao^a*^, Feng-Wu Bai^a^

^a^Key Laboratory of Microbial Metabolism, Joint International Research Laboratory of Metabolic & Developmental Sciences, School of Life Sciences and Biotechnology, Shanghai Jiao Tong University, Shanghai 200240, China

^b^Graduate School of Science, Technology and Innovation, Kobe University, Kobe, Japan; Engineering Biology Research Center, Kobe University, Kobe, Japan.

^c^SINOPEC Dalian Research Institute of Petroleum and Petrochemicals Co., Ltd., Dalian 116045, China.

*Corresponding author, Email addresses: xqzhao@sjtu.edu.cn.

**Table S1.** Strains and plasmids used in this work

| Strains or plasmids | Description | Source |
| --- | --- | --- |
| Strains |  |  |
| Cv310 | *Candida viswanathii* CICC 33310 | CICC |
| Ct1798 | *Candida tropicalis* CICC 1798 | CICC |
| AXT3K | *S. cerevisiae* AXT3K | Yeast Na^+^ Transport Assay Kit |
| AXT3K-pYES2-SOS1-998 | *S. cerevisiae* AXT3K-pYES2-*SOS1*-998 | Yeast Na^+^ Transport Assay Kit |
| AXT3K-pYES2 | *S. cerevisiae* AXT3K-pYES2 | Yeast Na^+^ Transport Assay Kit |
| Plasmids |  |  |
| pUC57-ARS-Hyg^R^ | Amp, ARS, Hyg^R^ | This study |
| pUC57-ARS-Nrs^R^ | Amp, ARS, Nrs^R^ | This study |
| pUCC001 | Hyg^R^ | Laboratory stock |
| pUC57-ARS-Nrs^R^-Cre | Amp, ARS, Nrs^R^, P*_ADH2_*-Cre-T*_CYC1_* | This study |
| pCashGem-Xyl2-URA3-LINEAR | Amp, P*_ENO1_*-Cas9-Geminin-T*_TEF1_*, P*_SNR52_*-gRNA | ^1^ |
| pCashGem-POX5-Hyg^R^-LINEAR | Amp, P*_ENO1_*-Cas9-Geminin-T*_TEF1_*, P*_SNR52_*-gRNA-POX5, Hyg^R^ | This study |
| pCashGem-KU70-Hyg^R^-LINEAR | Amp, P*_ENO1_*-Cas9-Geminin-T*_TEF1_*, P*_SNR52_*-gRNA-KU70, Hyg^R^ | This study |
| pCT-tRNA | Amp, P*_ACT1_*-Cas9-T*_TRP1_*, P*_TEF1_*-tRNA^Ala^-gRNA-T*_CYC1_*, Nrs^R^ | ^2^ |
| pXY1-Cas9 | Amp, P*_TDH1_*-Cas9-T*_TRP1_*, P*_TEF1_*-tRNA^Ala^-gRNA-T*_CYC1_*, Nrs^R^ | This study |
| pXY1-Cas9-POX5 | Amp, P*_TDH1_*-Cas9-T*_TRP1_*, P*_TEF1_*-tRNA^Ala^-POX5-T*_CYC1_*, Nrs^R^ | This study |
| pXY101-Cas9-POX5 | Amp, P*_TDH1_*-Cas9-T*_TRP1_*, P*_PGK1_*-tRNA^Ala^-POX5-T*_CYC1_*, Nrs^R^ | This study |
| pXY102-Cas9-POX5 | Amp, P*_TDH1_*-Cas9-T*_TRP1_*, P*_ENO2_*-tRNA^Ala^-POX5-T*_CYC1_*, Nrs^R^ | This study |
| pXY103-Cas9-POX5 | Amp, P*_TDH1_*-Cas9-T*_TRP1_*, P*_TDH3_*-tRNA^Ala^-POX5-T*_CYC1_*, Nrs^R^ | This study |
| pXY2-Cas9 | Amp, CaARS, Nrs^R^, P*_ENO1_*-Cas9-Geminin-T*_TEF1_*, P*_SNR52_*-gRNA | This study |
| pXY2-Cas9-POX5 | Amp, CaARS, Nrs^R^, P*_ENO1_*-Cas9-Geminin-T*_TEF1_*, P*_SNR52_*-gRNA-POX5 | This study |

**Table S1.** (Continued) Strains and plasmids used in this work

| Strains or plasmids | Description | Source |
| --- | --- | --- |
| pXY1-Cas9-sgRNA-Plus | Nrs^R^, P*_TDH1_*-Cas9-T*_TRP1_*, T*_TEF1_*-tRNA^Ala^-gRNA-tRNA^Ala^-gRNA-T*_CYC1_* | This study |
| pXY1-Cas9-sgRNA-FAT1-POX5 | Nrs^R^, P*_TDH1_*-Cas9-T*_TRP1_*, T*_TEF1_*-tRNA^Ala^-*POX5*-tRNA^Ala^-FAT1-T*_CYC1_* | This study |
| pUC57-*POX5*-UP-Hyg^R^-DW | Amp, Donor-*POX5*-UP-Hyg^R^-DW | This study |
| pUC57-*FAT1*-UP-Hyg^R^-DW | Amp, Donor-*FAT1*-UP-Hyg^R^-DW | This study |
| pUC57-ARS-Hyg^R^-P*_ADH1_*-GFP-T*_CYC1_* | Amp, CaARS, Hyg^R^, P*_ADH1_*-GFP-T*_CYC1_* | This study |
| pUC57-ARS-Hyg^R^-P*_AOX2_*-GFP-T*_CYC1_* | Amp, CaARS, Hyg^R^, P*_AOX2_*-GFP-T*_CYC1_* | This study |
| pUC57-ARS-Hyg^R^-P*_ADH2_*-GFP-T*_CYC1_* | Amp, CaARS, Hyg^R^, P*_ADH2_*-GFP-T*_CYC1_* | This study |
| pUC57-ARS-Hyg^R^-P*_PGK1_*-GFP-T*_CYC1_* | Amp, CaARS, Hyg^R^, P*_PGK1_*-GFP-T*_CYC1_* | This study |
| pUC57-ARS-Hyg^R^-P*_TDH1_*-GFP-T*_CYC1_* | Amp, CaARS, Hyg^R^, P*_TDH1_*-GFP-T*_CYC1_* | This study |
| pUC57-ARS-Hyg^R^-P*_ENO1_*-GFP-T*_CYC1_* | Amp, CaARS, Hyg^R^, P*_ENO1_*-GFP-T*_CYC1_* | This study |
| pUC57-ARS-Hyg^R^-P*_FBA1_*-GFP-T*_CYC1_* | Amp, CaARS, Hyg^R^, P*_FBA1_*-GFP-T*_CYC1_* | This study |
| pUC57-ARS-Hyg^R^-P*_TEF2_*-GFP-T*_CYC1_* | Amp, CaARS, Hyg^R^, P*_TEF2_*-GFP-T*_CYC1_* | This study |
| pXY1-Cas9-*POX4* | Amp, P*_TDH1_*-Cas9-T*_TRP1_*, P*_TEF1_*-tRNA^Ala^-*POX4*-T*_CYC1_*, Nrs^R^ | This study |
| pUC57-*POX4*-UP-Hyg^R^-GFP-DW | Amp, Donor-*POX4*-UP-Hyg^R^-GFP-DW | This study |
| pXY1-Cas9-rDNA | Amp, P*_TDH1_*-Cas9-T*_TRP1_*, P*_TEF1_*-tRNA^Ala^-rDNA-T*_CYC1_*, Nrs^R^ | This study |
| pUC57-rDNA-UP-Hyg^R^-GFP-DW | Amp, Donor-rDNA-UP-Hyg^R^-GFP-DW | This study |
| pUC57-ARS-Hyg^R^-P*_TDH1_*-*ENA1*-GFP-T*_CYC1_* | Amp, ARS, Hyg^R^, P*_TDH1_*-*ENA1*-linker-GFP-T*_CYC1_* | This study |
| pYES2-*g144** | Amp, *URA3*, P*_GAL1_*-*g144*-*T*_CYC1_* | This study |
| pXY1-Cas9-*G144* | Amp, P*_TDH1_*-Cas9-T*_TRP1_*, P*_TEF1_*-tRNA^Ala^-*G144*-T*_CYC1_*, Nrs^R^ | This study |
| pUC57-*g144*-UP-Hyg^R^-DW | Donor-*g144*-UP-Hyg^R^-DW | This study |
| pUC57-ARS-Hyg^R^-P*_NCP1_*-*G144*-T*_CYC1_* | Amp, ARS, Hyg^R^, P*_NCP1_*-*G144*-T*_CYC1_* | This study |
| pUC57-ARS-Hyg^R^-P*_FBA1_*-*G144*-T*_CYC1_* | Amp, ARS, Hyg^R^, P*_FBA1_*-*G144*-T*_CYC1_* | This study |

**Table S2. Primers used in this work**

| **Primer** | **Sequence** | **Description** |
| --- | --- | --- |
| TY-FP-pUC57-F | ggatccACTGGCCGTCGTTTTAC | For construction of pUC57-ARS-Hyg^R^ |
| TY-FP-pUC57-R | gaattcTGGTCATAGCTGTTTCCTG |  |
| FP-ARS-pUC57-F | AACATGGGATCCATTGCGTTG |  |
| PFBA1-F | TCTTACCCATCCCGGGTGTAGATCGATTAATAGTTGTTTTTGATTAG |  |
| PFBA1-R | GCAACGCAATggatcccatgttGTCTCACAGATTATTCCTCGGAGG |  |
| YH-HygR-F2 | CGATCTACACCCGGGATGGGTAAGAAGCC |  |
| YH-HygR-R2 | tgtcctcgagCTATTCCTTAGCTCTTGGTCTGGTGG |  |
| THygR-F | AAACGACGGCCAGTggatccgctcatctcgagggagatcactaaac |  |
| THygR-R | AGAGCTAAGGAATAGctcgaggacaataaaaagattcttg |  |
| NrsR-F | ttaggggcagggcatgctcatgtag |  |
| NrsR-R | atgggtaccactcttgacgac |  |
| FP-pUC57-NrsR-F | gtgtcgtcaagagtggtacccatCCCGGGTGTAGATCGATTAATAG |  |
| FP-pUC57-NrsR-R | ctacatgagcatgccctgcccctaactcgaggacaataaaaagattcttg |  |
| Ct1798ADH2p-F | GTAAAACGACGGCCAGTggatccAAATTAAGATTTGGTGGGGATGG | For construction of pUC57-ARS-Nrs^R^-Cre |
| Ct1798ADH2p-R | GTCTGTCTCTGAACATGTCCATGCTTATTGTAGTTTTGTAAAAGTCAATTG |  |
| Cre-F | CTAGTCACCATCTTCCAACAATCTG |  |
| Cre-R | ATGGACATGTTCAGAGACAGACA |  |
| CYC1t-F | CAGATTGTTGGAAGATGGTGACTAGTCATGTAATTAGTTATGTCACGCTTAC |  |
| CYC1t-R | ccctcgagatgagcCCGCGGGCAAATTAAAGCCTTCGAGCG |  |
| TY-FP-pUC57-HygR-R | CCGCGGgctcatctcgagggagatcactaaac |  |
| FP-pGEM-F1 | aggtaccttatgtaaggcggc | For construction of pCashGem-*POX5*-Hyg^R^-LINEAR |
| FP-pGEM-POX5-R | TGGTGTTTTCTGGTGGGTTCgaaataaatgtatcttgttggaaacgaacctg |  |
| sgRNA-POX5-F | GAACCCACCAGAAAACACCAgttttagagctagaaatagcaag |  |
| sgRNA-POX5-R | CCTTGGTGTTTTCTGGTGGGTTCgatgctcgataaaaaaactcgag |  |
| POX5-UP-F | GAACCCACCAGAAAACACCAAGGGAAGTCACCATGCCACCATC |  |

**Table S2.** (Continued) Primers used in this work

| **Primer** | **Sequence** | **Description** |
| --- | --- | --- |
| POX5-g129-g546-UP-R2 | GGAAACCAAAACCAAACTTGGAC | For construction of pCashGem-*POX5*-Hyg^R^-LINEAR |
| POX5-loxP-HygR-F | CCAAGTTTGGTTTTGGTTTCCTACCGTTCGTATAGCATACATTATACGAAGTTATGTCTCACAGATTATTCCTCGGAG |  |
| POX5-loxp-HygR-R | GTGCTCTATTAACTGGACAAGTACCGTTCGTATAATGTATGCTATACGAAGTTATgctcatctcgagggagatcac |  |
| POX5-g129-g546-DW-F | CTTGTCCAGTTAATAGAGCACTAGG |  |
| POX5-DW-R | gccgccttacataaggtacctGTAGGACGAGTTAGCAAGCAC |  |
| YZ-POX5-g129-g546-F | GTCTACGCCAGATTGAAGGTC |  |
| YZ-POX5-g129-g546-R | AGTATCATACTTGCCACACGC |  |
| CX-loxp-HygR-F | CTATTGTTAGATCCGGTGCTGG |  |
| CX-loxp-HygR-R | GTTTCCAATTCTTTATCCCTGATCC |  |
| FP-pGEM-KU70-R | TCGTGTACAACAACAAGTCGgaaataaatgtatcttgttggaaacgaacctg | For construction of pCashGem-*KU70*-Hyg^R^-LINEAR |
| sgRNA-KU70-F | CGACTTGTTGTTGTACACGAgttttagagctagaaatagcaag |  |
| sgRNA-KU70-R | CCTTCGTGTACAACAACAAGTCGgatgctcgataaaaaaactcgag |  |
| KU70-UP-F2 | CGACTTGTTGTTGTACACGAAGGATGAGTTGGAACACACAGGAC |  |
| Ku70-UP-R2 | TGGCTCCTCTTCAAATAGTTTGTG |  |
| Ku70-loxp-HygR-F | CACAAACTATTTGAAGAGGAGCCATACCGTTCGTATAGCATACATTATACGAAGTTATGTCTCACAGATTATTCCTCGGAG |  |
| Ku70-loxp-HygR-R | AATTTCGAGGAGTTGGTGGTCTACCGTTCGTATAATGTATGCTATACGAAGTTATgctcatctcgagggagatcac |  |
| Ku70-DW-F | GACCACCAACTCCTCGAAATTG |  |
| Ku70-DW-R2 | cggccgccttacataaggtacctTCATTGTTTCTGCCTACTGTCTAAG |  |
| YZ-Ku70-F | GCTCAGTTGGTAGAGCATTAGAC |  |
| YZ-Ku70-R | ACGAGAATGAAGTGTATGGACATC |  |

**Table S2.** (Continued) Primers used in this work

| **Primer** | **Sequence** | **Description** |
| --- | --- | --- |
| sgRNA-POX5-F | ccaGAACCCACCAGAAAACACCA | For construction of pXY1-Cas9-OK,  pXY1-Cas9-POX5, pXY101-Cas9-POX5,  pXY102-Cas9-POX5,  pXY103-Cas9-POX5,  pXY2-Cas9-OK,  pXY2-Cas9-POX5,  pXY1-Cas9-sgRNA-Plus,  pXY1-Cas9-sgRNA-*FAT1-POX5*,  pUC57-*POX5*-UP-Hyg^R^-DW,  pUC57-*FAT1*-UP-Hyg^R^-DW |
| sgRNA-POX5-R | aacTGGTGTTTTCTGGTGGGTTC |  |
| FP-pCT-F2 | ggtattctgggcctccatgtc |  |
| tRNA-Ala-F | aaacaaagagcttaaaatgggcg |  |
| TDH1p-F2 | gttatgtgagcggccgcggtaccTCACAAGCGATCAACTTATGGTAG |  |
| TDH1p-R2 | tatccatcggatccctcgagcTGTTTAAATTCTTTAATTGAGGGATG |  |
| PGK1p-F2 | gacatggaggcccagaataccTTGTTGTGCAATTGTCCAGTG |  |
| PGK1p-R2 | cacgcccattttaagctctttgtttTCTGAATAGGCAATTGATAAATTTAC |  |
| ENO2p-F2 | gacatggaggcccagaataccGAGGTGAATATTTTAGTGGTTCCAATC |  |
| ENO2p-R2 | cacgcccattttaagctctttgtttTGTGCGATTGGCAGGGGGGA |  |
| CtTDH3p-F | gacatggaggcccagaataccTTTGGTTCACACCACTACCG |  |
| CtTDH3p-R | cacgcccattttaagctctttgtttTGTTAAAATTTAATTTGTAAGTGATTTG |  |
| sgRNA-POX5-F3 | ttcGAACCCACCAGAAAACACCA |  |
| sgRNA-POX5-R3 | aacTGGTGTTTTCTGGTGGGTTC |  |
| FP-CashGem-F | atcaggtgcaccttccctgcagggtgagcgcaacgcaatTAATG |  |
| FP-CashGem-R | ctgccctcttccagctggatcccttcctcgctcactgactcg |  |
| pCashGem-BOX-F | tgcagggaaggtgcacctgat |  |
| pCashGem-BOX-R | ggatccagctggaagagggcag |  |
| pXY2-sg-OK-F2 | gaaataaatgtatcttgttggaaacg |  |
| pXY2-sg-OK-R2 | gttttagagctagaaatagcaag |  |
| Don-BspQI-F | cgtttccaacaagatacatttatttcggaagagctcgcgagctcttccgttttagagctagaaatagcaag |  |
| Don-BspQI-R | cttgctatttctagctctaaaacggaagagctcgcgagctcttccgaaataaatgtatcttgttggaaacg |  |
| Double-sgBox-F | gttttagagctagaaatagcaag |  |
| FP-pCT-F | gtcccattcgccatgccgaag |  |
| sgRNA-BOX-F | cttcggcatggcgaatgggactaaaatgggcgtgtggcgtag |  |
| Double-sgBox-R | tggacgagataagaatcgaactc |  |
| Double-FAT1-sgRNA-F | agttcgattcttatctcgtccaGGAGTGGTACAAGGGCATGGgttttagagctagaaatagcaag |  |
| Double-POX5-sgRNA-R | cttgctatttctagctctaaaacTGGTGTTTTCTGGTGGGTTCtggacgagataagaatcgaactc |  |

**Table S2.** (Continued) Primers used in this work

| **Primer** | **Sequence** | **Description** |
| --- | --- | --- |
| TEF2p-F | GTAAAACGACGGCCAGTggatccTTGTTCCAGTACAAGTGCAAGG | For construction of pUC57-ARS-Hyg^R^-P*_ADH1_*-GFP-T*_CYC1_*,  pUC57-ARS-Hyg^R^-P*_AOX2_*-GFP-T*_CYC1_*,  pUC57-ARS-Hyg^R^-P*_PCK1_*-GFP-T*_CYC1_*, pUC57-ARS-Hyg^R^-P*_PGK1_*-GFP-T*_CYC1_*,  pUC57-ARS-Hyg^R^-P*_TDH1_*-GFP-T*_CYC1_*,  pUC57-ARS-Hyg^R^-P*_ENO1_*-GFP-T*_CYC1_*,  pUC57-ARS-Hyg^R^-P*_FBA1_*-GFP-T*_CYC1_*,  pUC57-ARS-Hyg^R^-P*_TEF2_*-GFP-T*_CYC1_* |
| TEF2p-R | CAATTCTTCACCTTTAGACATGATTGATTATTTCTTTAGATGTGTAG |  |
| ADH1p-F | GTAAAACGACGGCCAGTggatccGCTCACAGATAGCGACTCC |  |
| ADH1p-R | CAATTCTTCACCTTTAGACATGAATGCAAGAAAATATGCAAGACTG |  |
| AOX2p-F | GTAAAACGACGGCCAGTggatccAACTCCGCTTCTTCGTGAC |  |
| AOX2p-R | CAATTCTTCACCTTTAGACATGGTTGGTTGGGTGATTGGTAATG |  |
| ENO1p-F | GTAAAACGACGGCCAGTggatccGAGGTGAATATTTTAGTGGTTCCAATC |  |
| ENO1p-R | CAATTCTTCACCTTTAGACATTGTGCGATTGGCAGGGGGGA |  |
| FBA1p-F | GTAAAACGACGGCCAGTggatccGACACAAACCTCCTCAGGTC |  |
| FBA1p-R | CAATTCTTCACCTTTAGACATTGTTGGATGATTTGTAGATAAGGTAG |  |
| PCK1p-F | GTAAAACGACGGCCAGTggatccAGTGCGATCACCTACTTTGG |  |
| PCK1p-R | CAATTCTTCACCTTTAGACATATTGAATAGTTAAAGTGTAATGGAG |  |
| PGK1p-F | GTAAAACGACGGCCAGTggatccTTGTTGTGCAATTGTCCAGTG |  |
| PGK1p-R | CAATTCTTCACCTTTAGACATTCTGAATAGGCAATTGATAAATTTAC |  |
| TDH1p-F | GTAAAACGACGGCCAGTggatccTCACAAGCGATCAACTTATGGTAG |  |
| TDH1p-R | CAATTCTTCACCTTTAGACATTGTTTAAATTCTTTAATTGAGGGATG |  |
| GFP-F2 | ATGTCTAAAGGTGAAGAATTGTTTACTG |  |
| FP-TYCZ-F | GAACGTAACCTTCAGGCATAGC | For assay of Homologous recombination activity |
| FP-TYCZ-R | GAAGATGGTTCTGTTCAATTGGC |  |
| TYCZ-F | GCTATGCCTGAAGGTTACGTTC |  |
| TYCZ-R | GCCAATTGAACAGAACCATCTTC |  |
| POX4-sgRNA-F | ccaGTGTCTTCTTGACTTTCAAC | For construction of pXY1-Cas9-*POX4*, pUC57-*POX4*-UP-Hyg^R^-GFP-DW |
| POX4-sgRNA-R | aacGTTGAAAGTCAAGAAGACAC |  |
| POX4-UP-F | ATTGTCCATGTGAAGGCAGTACC |  |
| POX4-UP-R | GTCAGGACCTTGTGATACACTAAC |  |

**Table S2.** (Continued) Primers used in this work

| **Primer** | **Sequence** | **Description** |
| --- | --- | --- |
| POX4-lox-HygB-F | GTTAGTGTATCACAAGGTCCTGACTACCGTTCGTATAGCATACATTATACGAAGTTATGTCTCACAGATTATTCCTCGGAG | For construction of pXY1-Cas9-*POX4*, pUC57-*POX4*-UP-Hyg^R^-GFP-DW |
| POX4-lox-HygB-R | CCATAAGTTGATCGCTTGTGATACCGTTCGTATAATGTATGCTATACGAAGTTATgctcatctcgagggagatcac |  |
| GFP-BOX-F | TCACAAGCGATCAACTTATGG |  |
| CYC1t-R2 | CACTCTCTTCTATTCTTACTTGGAGCAAATTAAAGCCTTCGAGCG |  |
| POX4-DW-F | TCCAAGTAAGAATAGAAGAGAGTGAC |  |
| POX4-DW-R | AGTACAAGTACGGGAATAGATGCAC |  |
| YZ-POX4-F | TCTGAGCTTGTCCGTACGAG |  |
| YZ-POX4-R | ACGACAAGACGAAGTAATTAGC |  |
| rDNA-sgRNA-F | ccaGTTTATGGTTAAGACTACGA | For construction of pXY1-Cas9-rDNA,  pUC57-rDNA-UP-Hyg^R^-GFP-DW |
| rDNA-sgRNA-R | aacTCGTAGTCTTAACCATAAAC |  |
| rDNA-UP-F | GTAAAACGACGGCCAGTggatccAATGATCCTTCCGCAGGTTC |  |
| rDNA-UP-R | CTCACCAGGTCCAGACACAATAAG |  |
| rDNA-DW-F | CAACCAAGCCCAAGGTTCAAC |  |
| rDNA-DW-R | GAAACAGCTATGACCAgaattcATCTGGTTGATCCTGCCAGTAG |  |
| rDNA-loxp-HygB-F | CTTATTGTGTCTGGACCTGGTGAGTACCGTTCGTATAGCATACATTATACGAAGTTATGTCTCACAGATTATTCCTCGGAG |  |
| CYC1t-R3 | GTTGAACCTTGGGCTTGGTTGGCAAATTAAAGCCTTCGAGCG |  |
| YZ-rDNA-F | GATGCGAGAACCAAGAGATCC |  |
| YZ-rDNA-R | TTGGCCATGTTCGACCTGTG |  |
| FBA1p-F | GTAAAACGACGGCCAGTggatccGACACAAACCTCCTCAGGTC | For construction of pUC57-ARS-Hyg^R^-P*_FBA1_*-g144-GFP-T*_CYC1_* |
|  |  |  |
| linker-GFP-F | ggtgacggtgctggtttaattaacATGTCTAAAGGTGAAGAATTGTTTACTG |  |
| g144-F | ATGATCTTGGTGTTGATCATCAG |  |
| g144-R | gttaattaaaccagcaccgtcaccGACAACCATCGTGTTGTCCC |  |
| g144-F2 | TAAGCTTGGTACCGAGCTCGGATCCATGATCTTGGTGTTGATCATTTCC | For construction of pYES2-g144* |
| g144-R2 | TCTAGATGCATGCTCGAGCGGCCGCTCAAACGACCATGGTATTGTCAC |  |

**Table S2.** (Continued) Primers used in this work

| **Primer** | **Sequence** | **Description** |
| --- | --- | --- |
| g144-sgRNA-F | ccaCAAGCCAAGGATTCTCTCCA | For construction of pXY1-Cas9-g144,  pUC57-g144-UP-Hyg^R^-DW |
| g144-sgRNA-R | aacTGGAGAGAATCCTTGGCTTG |  |
| g144-UP-F | AAACGACGGCCAGTggatccCCTCCGATGCCTATAGAATCTC |  |
| g144-UP-R | CCCAAGATGTTACCAATCACG |  |
| g144-loxp-HygR-F | CGTGATTGGTAACATCTTGGGTACCGTTCGTATAGCATACATTATACGAAGTTATGTCTCACAGATTATTCCTCGGAG |  |
| g144-loxp-HygR-R | CGTAGCCAATACCAATTGCTGTACCGTTCGTATAATGTATGCTATACGAAGTTATgctcatctcgagggagatcac |  |
| g144-DW-F | CAGCAATTGGTATTGGCTACG |  |
| g144-DW-R | AACAGCTATGACCAgaattcCGCAAGATCAAGGATACAACAC |  |
| YZ-g144-F | TGTTGTCCCTGCTGAACGAG |  |
| YZ-g144-R | ACTTGGTCAGCGGTCTTTCC |  |
| NCP1p-F | GTAAAACGACGGCCAGTggatccCGTTGGTTGAGTTTTCTCCG | For construction of pUC57-ARS-Hyg^R^-P*_NCP1_*-g144-T*_CYC1,_* pUC57-ARS-Hyg^R^-P*_FBA1_*-g144-T*_CYC1_* |
| NCP1p-R | GGTGAGGAGGAGGATGAGGAA |  |
| FBA1p-R | TGTTGGATGATTTGTAGATAAGGTAG |  |
| g144-F2 | CAATTCCTCATCCTCCTCCTCACCATGCGTTTCGTAACTGCTCATC |  |
| g144-R2 | CTTCTATCTACGACAATAGGACCTAGACAACCATCGTGTTGTCC |  |
| CYC1t-F2 | GTCCTATTGTCGTAGATAGAAGTCATGTAATTAGTTATGTCACGCTTAC |  |

Table S3. Amino acid site differences between g144 and Ena1

| Residues of g144 | Residues of Ena1 |
| --- | --- |
| 193 K | 307 Q |
| 203 I | 317 V |
| 206 I | 320 V |
| 209 V | 323 I |
| 397 E | 511 D |
| 402 A | 516 Q |
| 426 E | 540 D |
| 432 Y | 546 F |
| 454 K | 568 D |
| 470 G | 584 R |
| 491 P | 605 K |
| 492 L | 606 M |
| 495 A | 609 D |
| 497 V | 611 I |
| 498 K | 612 A |
| 524 S | 638 D |
| 525 A | 639 S |
| 529 Q | 643 D |
| 532 Q | 646 L |
| 538 V | 652 I |
| 641 D | 755 E |
| 648 K | 761 Q |
| 813 G | 927 A |
| 870 S | 984 V |
| 879 T | 993 A |
| 887 K | 1001 Q |
| 931 V | 1045 A |
| 943 V | 1057 A |
| 946 V | 1060 M |
| 966 S | 1080 F |


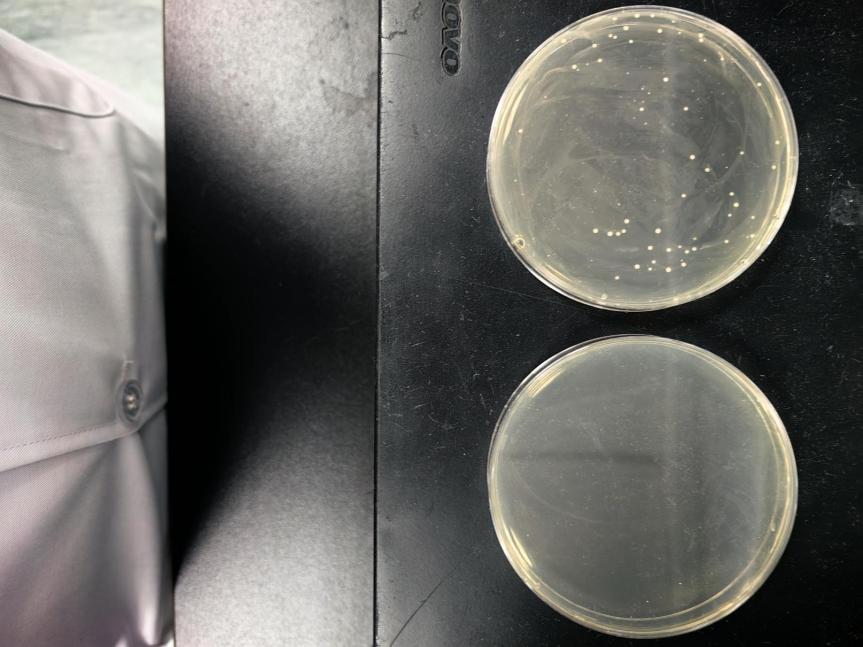


Figure S1. Virulence test of Cas9. The pXY1-Cas9-*POX5* was transformed into Cv310 (left plate) and Cv310Δ*KU70* (right plate), respectively.

*
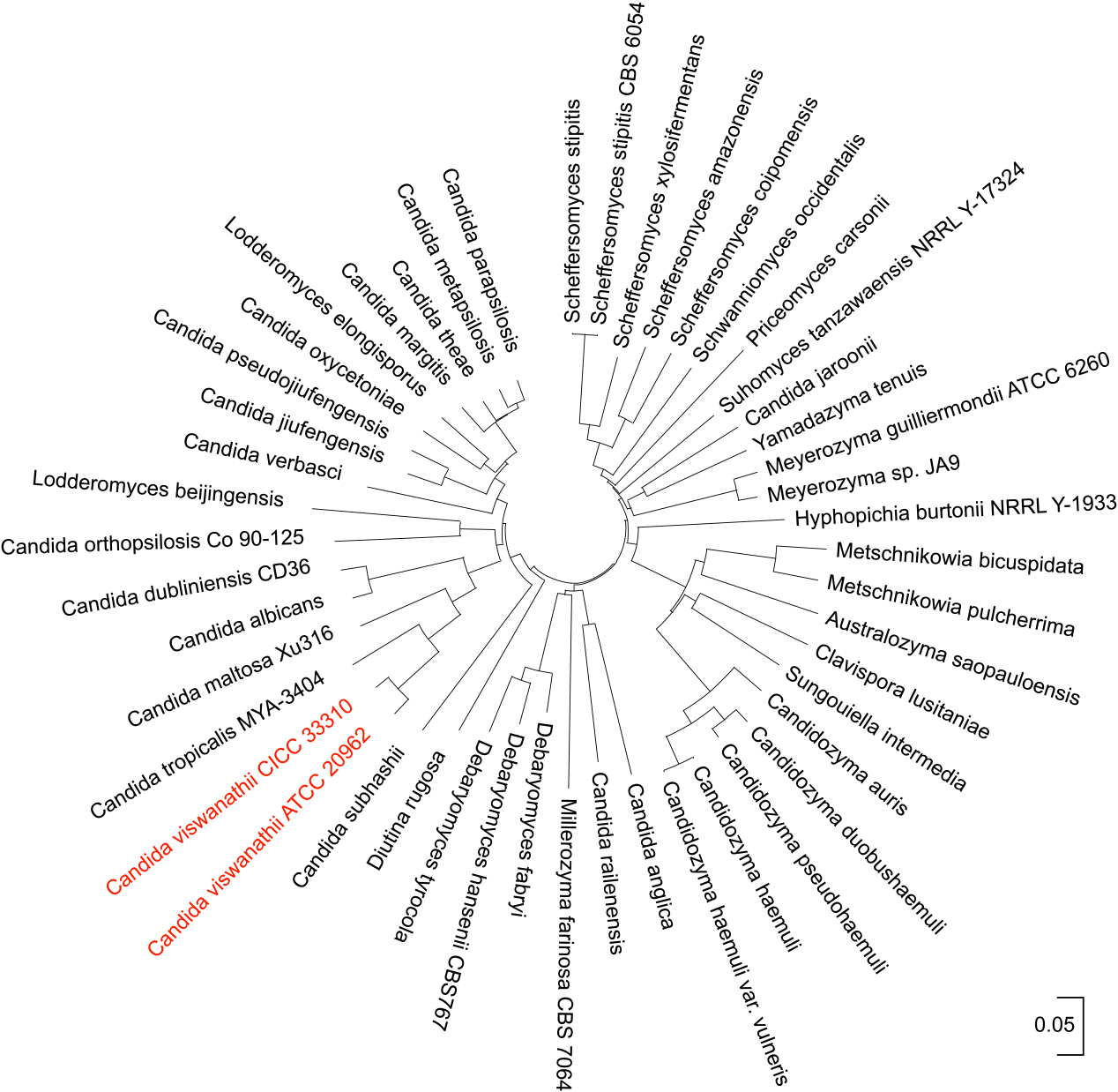
*

Figure S2. Phylogenetic tree analysis of g144. The 48 Ena1 protein sequences were obtained from the UniProt database, and the alignment was visualized using MEGA.


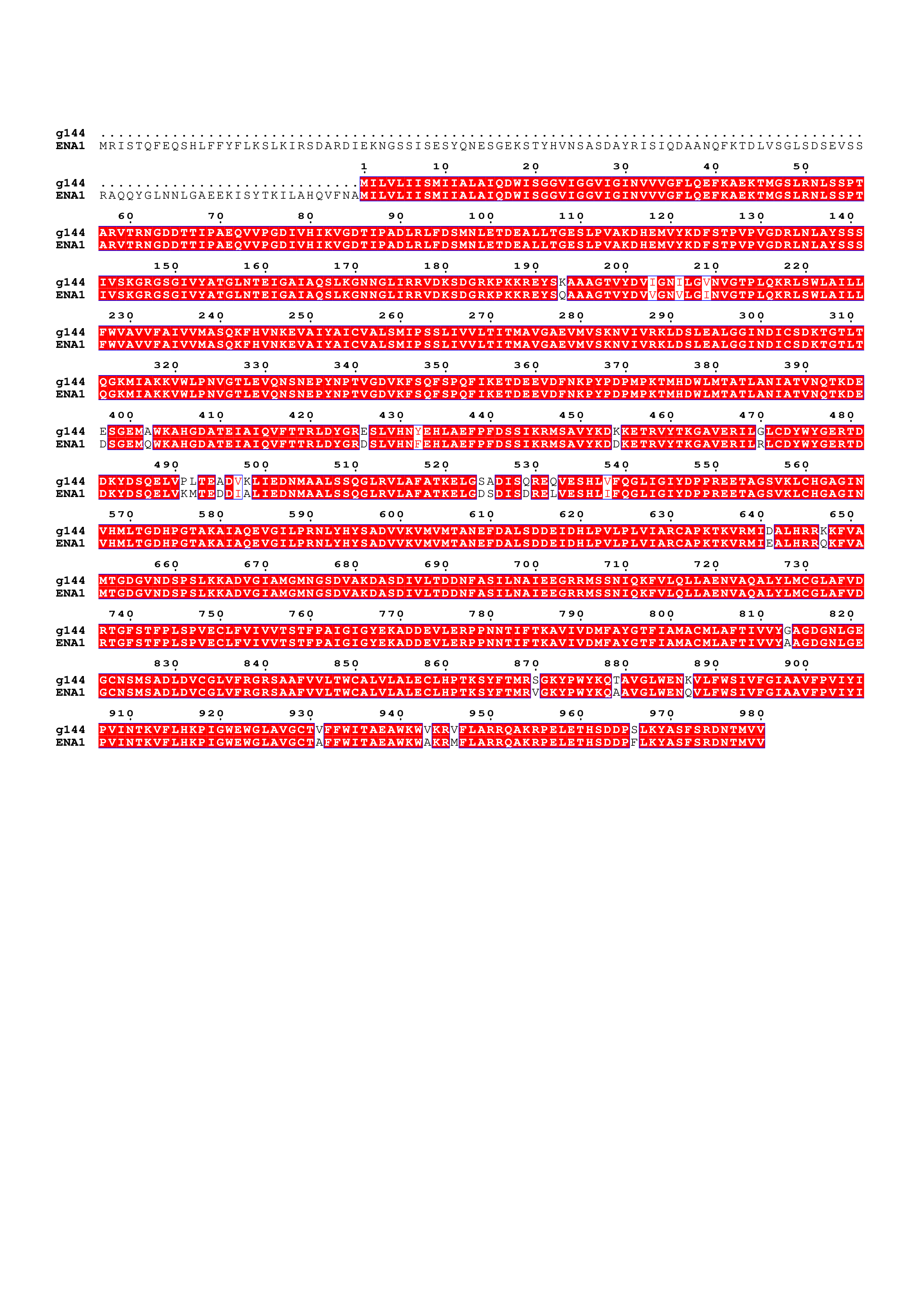


Figure S3. Amino acid sequence alignment between g144 and Ena1, generated using Clustal Omega (<https://www.ebi.ac.uk/Tools/msa/clustalo/>).


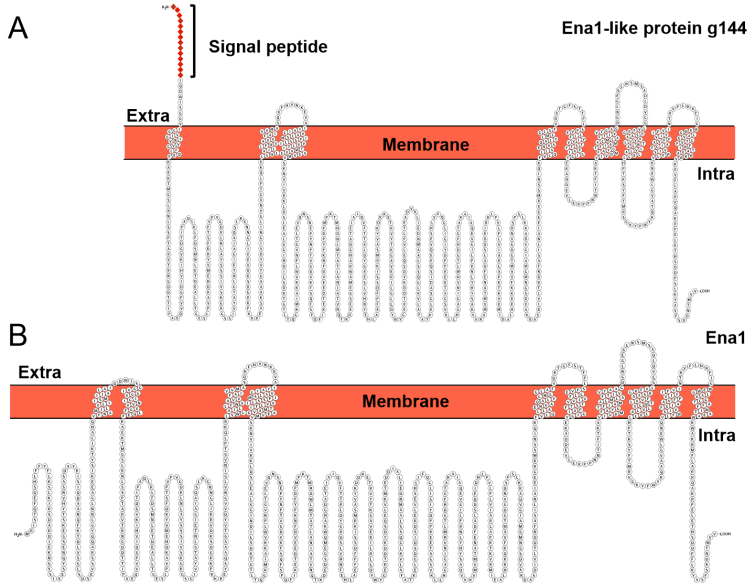


Figure S4. Visualisation of g144 and Ena1 transmembrane structure and localisation, generated using Protter (https://wlab.ethz.ch/protter/)^3^.


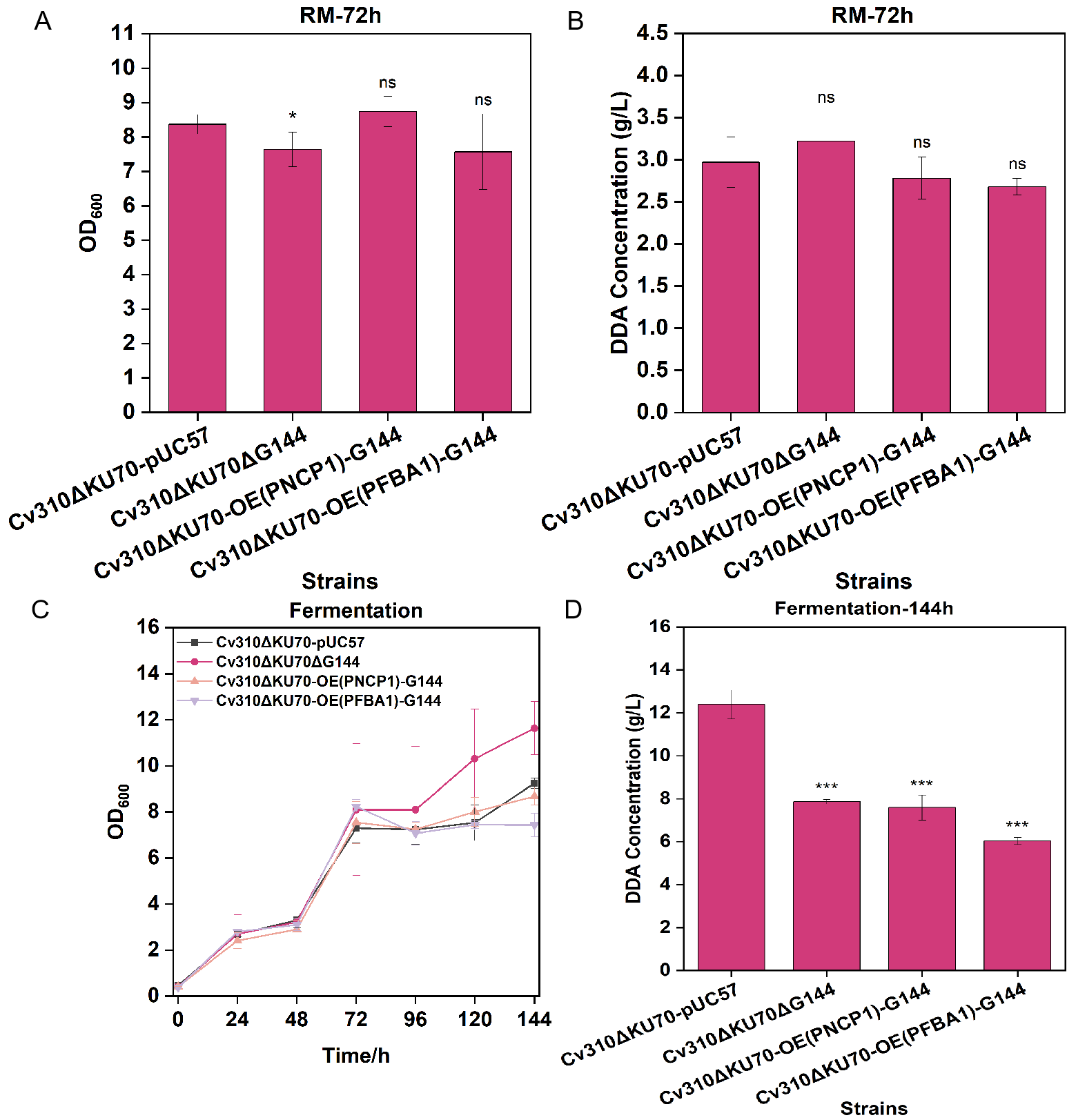


Figure S5. Phenotypic analysis of g144 recombinant strains cultured in different media. (A-B) OD_600_ and DDA production measured at 72 h in RM, (C-D) OD_600_ and DDA production under fermentation medium conditions.

**References**

1. Ploessl, D.; Zhao, Y.; Cao, M.; Ghosh, S.; Lopez, C.; Sayadi, M.; Chudalayandi, S. Severin, A.; Huang, L.; Gustafson, M.; Shao, Z., A repackaged CRISPR platform increases homology-directed repair for yeast engineering. *Nature Chemical Biology* **2021,** *18* (1), 38-46.

2. Lombardi, L.; Oliveira-Pacheco, J.; Butler, G., Plasmid-Based CRISPR-Cas9 Gene Editing in Multiple *Candida* Species. *mSphere* **2019,** *4* (2), 0-0.

3. Omasits, U.; Ahrens, C. H.; Müller, S.; Wollscheid, B., Protter: interactive protein feature visualization and integration with experimental proteomic data. *Bioinformatics* **2014,** *30* (6), 884-886.
